# A Case of Laryngeal Tuberculosis, Endobronchial Tuberculosis and Pulmonary Tuberculosis Coexistent in an Immunocompetent Host

**DOI:** 10.7759/cureus.10713

**Published:** 2020-09-29

**Authors:** Akshay Avula, Sam Ngu, Wissam Mansour, Dhineshreddy Gurala, Rabih Maroun

**Affiliations:** 1 Internal Medicine, Northwell Health-Staten Island University Hospital, Staten Island, USA; 2 Pulmonary and Critical Care Medicine, Northwell Health-Staten Island University Hospital, Staten Island, USA

**Keywords:** tuberculosis, laryngeal tuberculosis, endobronchial tuberculosis

## Abstract

Historically associated with poor prognosis seen in advanced disease, laryngeal tuberculosis (LTB) now represents only 1% of all cases of tuberculosis (TB). The incidence of LTB has decreased drastically with the introduction of anti-tubercular drugs. LTB can be primary or secondary to pulmonary tuberculosis. LTB can mimic laryngeal cancer. We present a case of primary laryngeal TB with descending tracheobronchial spread in an immunocompetent 71-year-old female who developed progressive dysphonia over several months with unintentional weight loss and non-productive cough. Non-contrast enhanced computed tomography (CT) revealed clustering of subcentimeter stellate nodules in the right upper lung field with an enlarging ground-glass opacity in the right lower lung but did not show structural abnormalities within the neck. Positron emission tomography (PET) showed pathologic fluorodeoxyglucose (FDG) uptake within the larynx and trachea with extension into the left mainstream bronchus as well as the proximal left upper and lower lobe bronchi. Diffuse standardized uptake value (SUV) was greatest in the larynx (20.5). Polymerase chain reaction (PCR) on bronchoscope sputum specimen confirmed Mycobacterium tuberculosis. Findings were consistent with primary laryngeal TB with endobronchial extension. She was started on a four-drug regimen comprising of isoniazid, rifampin, ethambutol, and pyrazinamide with a good response. Her close contacts were treated as well. This case highlights the unusual spread of primary laryngeal TB in an immunocompetent host. Early diagnosis can limit adverse complications and unnecessary exposure to healthcare workers. To our knowledge, this is the first case of primary LTB with proximal spread to the tracheobronchial and pulmonary tuberculosis.

## Introduction

The incidence of tuberculosis (TB) in the United States has been steadily declining since the advent of antituberculous medications with the lowest ever reported at 2.8 per 100,000 persons in 2018. Similarly, the incidence of laryngeal tuberculosis has been slowly declining. Most modern cases originate outside North America with developing countries having the highest incidence [1]. Highly contagious, close contact with an infected individual can instigate the purified protein derivative (PPD) conversion rate in 30-62.5% of cases. Thus, pose a serious public health concern. Here we present a rare case of primary laryngeal TB with descending tracheobronchial involvement in an immunocompetent elderly female retired nurse who developed progressive dysphonia over several months with unintentional weight loss, and non-productive cough. To our knowledge, this is the first case that is being reported of primary laryngeal tuberculosis (LTB) with proximal spread to the tracheobronchial and pulmonary tuberculosis.

## Case presentation

A 71-year-old female retired nurse with known chronic obstructive pulmonary disease presented with dysphonia and hoarseness of voice for five months. She also complained of recent onset of productive cough with clear sputum and 10% unintentional weight loss. She denied fevers, night sweats, or hemoptysis. As a nurse, she had multiple close contact exposures to individuals with active TB but was compliant with the use of appropriate personal protective equipment. She had consistently tested negative throughout her nursing career with annual Mantoux testing. Her travel history includes the Caribbean and Western and Central Europe. She is an ex-smoker in her teenage years.

**Figure 1:**
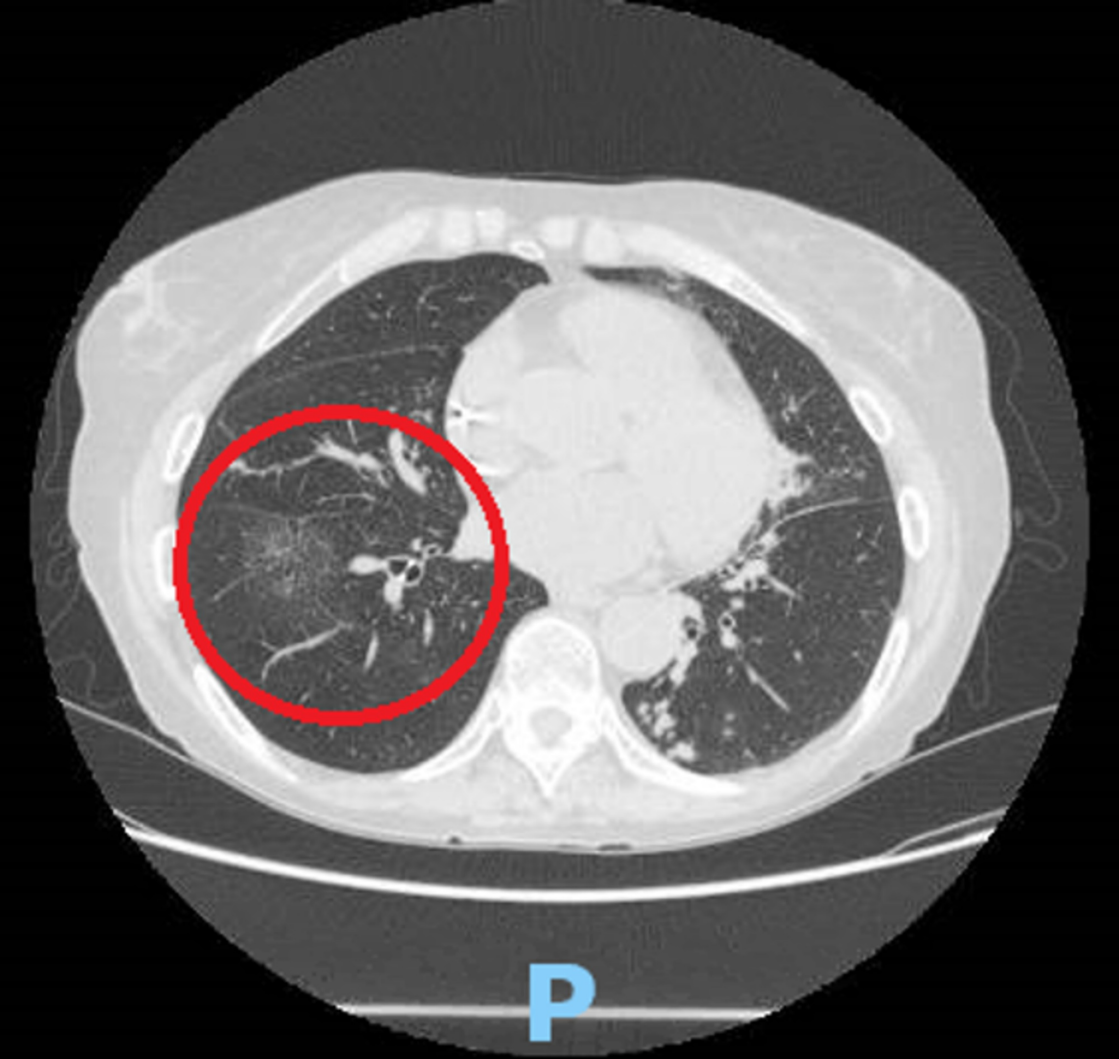
Non-contrast computed tomography of the chest, axial view, showing a new ground-glass opacity of the right lower lung field (red circle).

**Figure 2:**
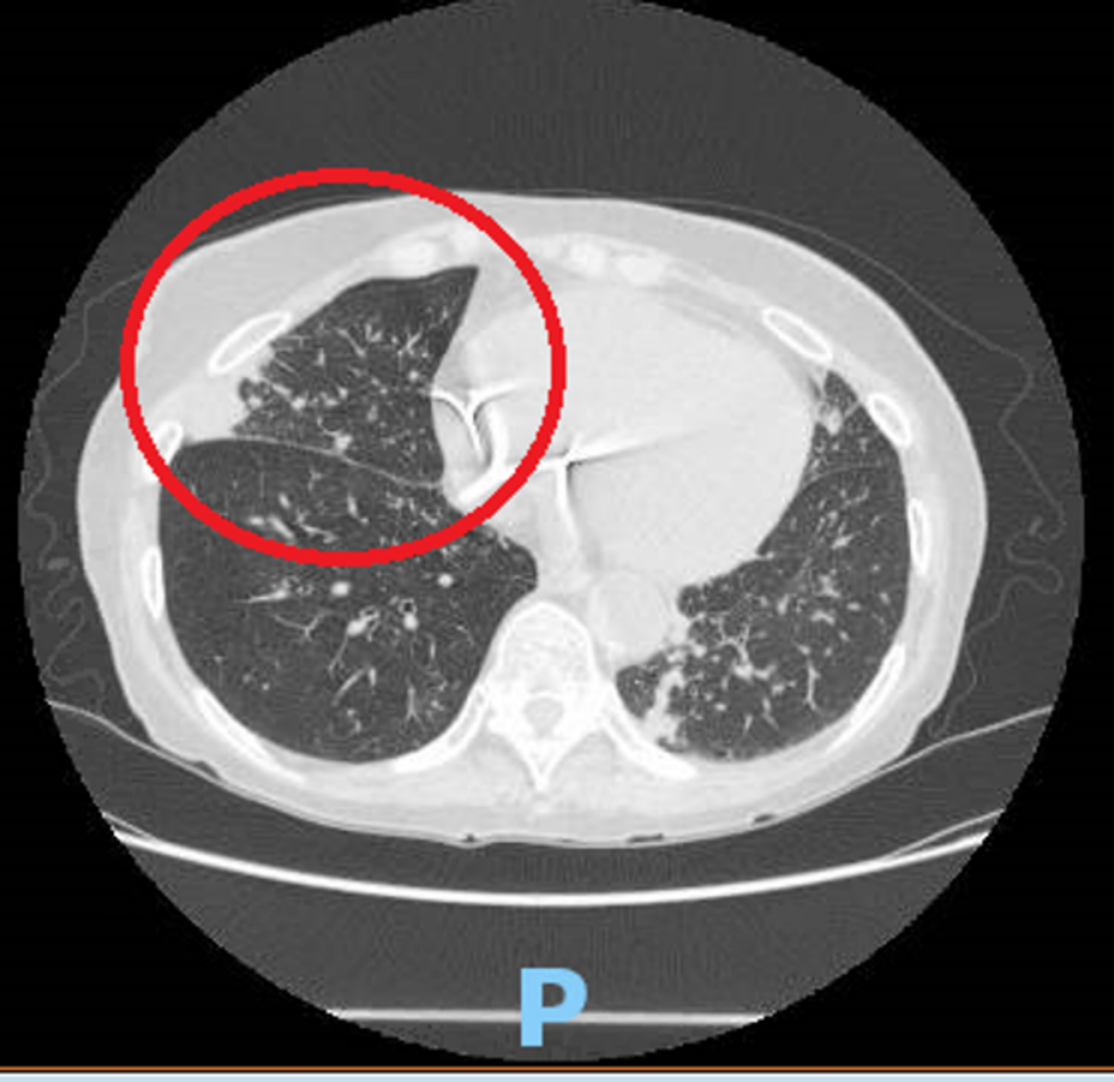
Non-contrast computed tomography of the chest, axial view, showing worsening pleural-based opacity with branching opacification within the inferior segment of the right lung (red circle).

Due to her complaints of dysphonia, she was referred by her primary doctor to otolaryngology, pulmonary and gastroenterology subspecialties. She underwent non-contrast-enhanced computed tomography (CT) that revealed clustering of subcentimeter stellate nodules in the right upper lung field with a ground-glass opacity in the right lower lung but did not show structural abnormalities within the neck (Figures 1, 2).

**Figure 3:**
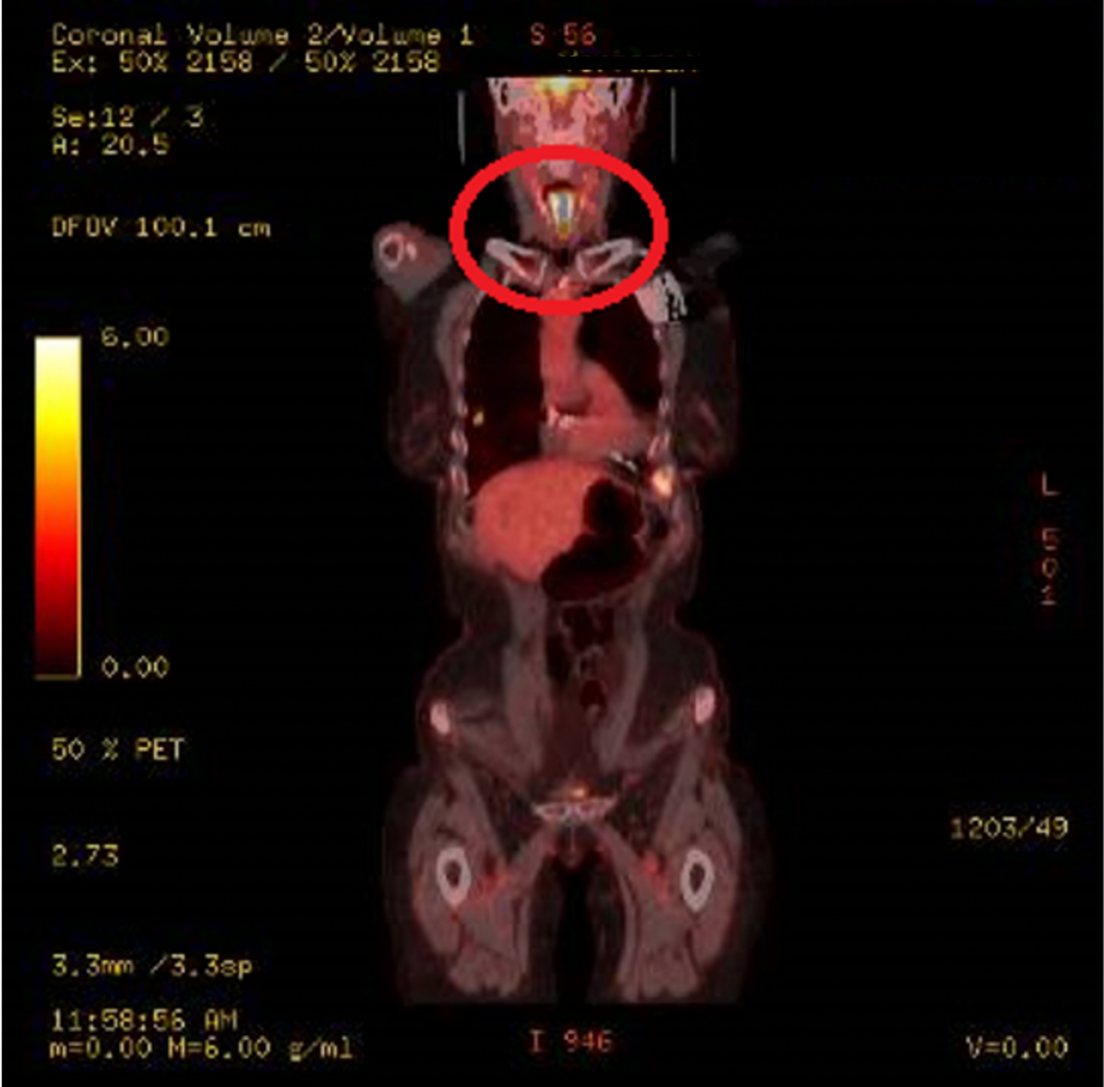
Positron emission tomography (PET) scan showing pathologic fluorodeoxyglucose (FDG) uptake within the larynx and trachea with extension into the left mainstream bronchus as well as the proximal left upper and lower lobe bronchi. The involvement of bilateral cervical chain lymph nodes was also noted. Diffuse standardized uptake value (SUV) was greatest in the larynx (20.5) (red circle).

Positron emission tomography (PET) showed pathologic fluorodeoxyglucose (FDG) uptake within the larynx and trachea with extension into the left mainstream bronchus as well as the proximal left upper and lower lobe bronchi. The involvement of bilateral cervical chain lymph nodes was also noted. Diffuse standardized uptake value (SUV) was greatest in the larynx (20.5) (Figure 3).

**Figure 4:**
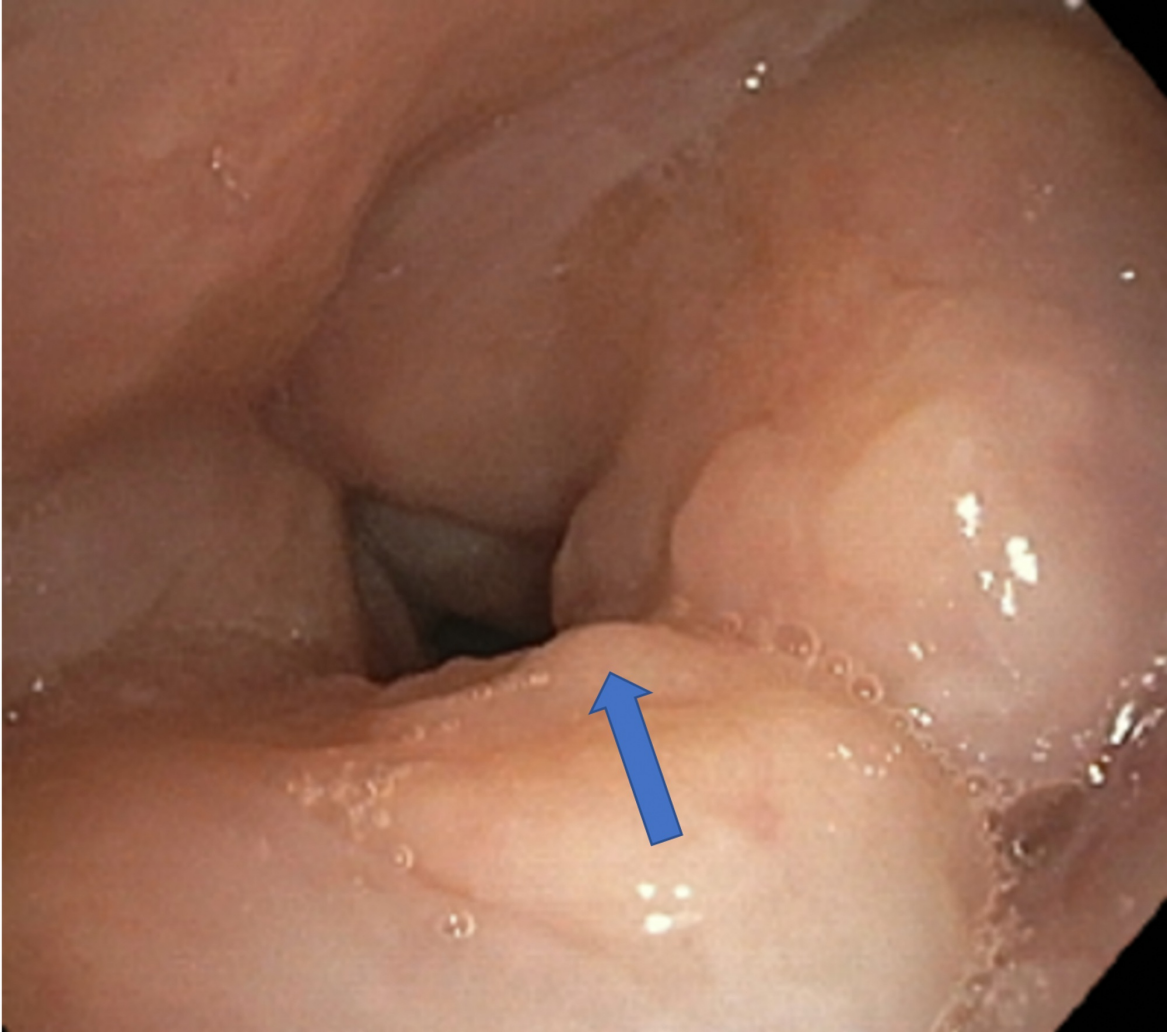
Bronchoscope imaging showing edematous supraglottic mucosa with normal vocal cords.

**Figure 5:**
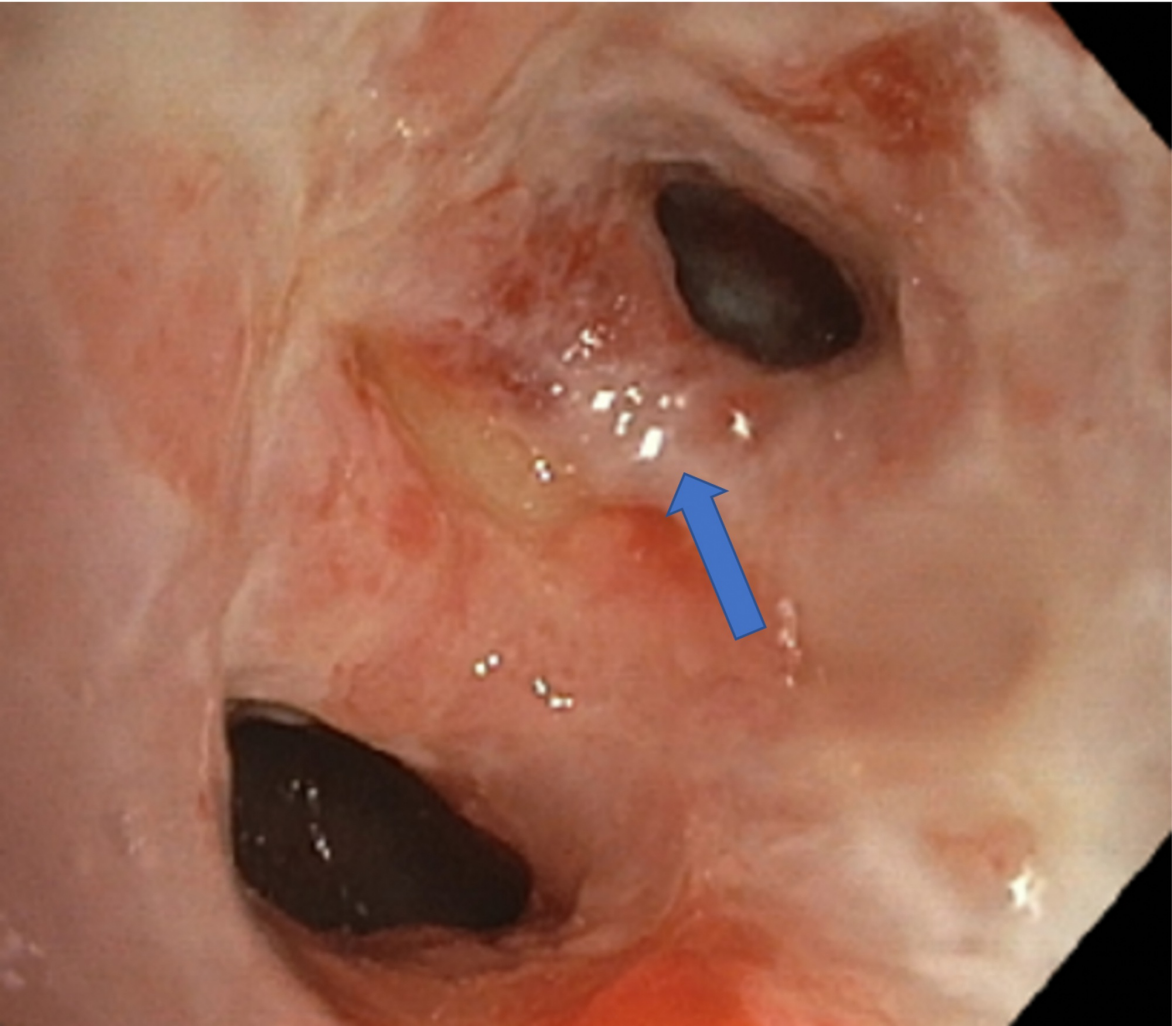
Bronchoscope imaging showing ulcerative lesion on the first carina.

**Figure 6:**
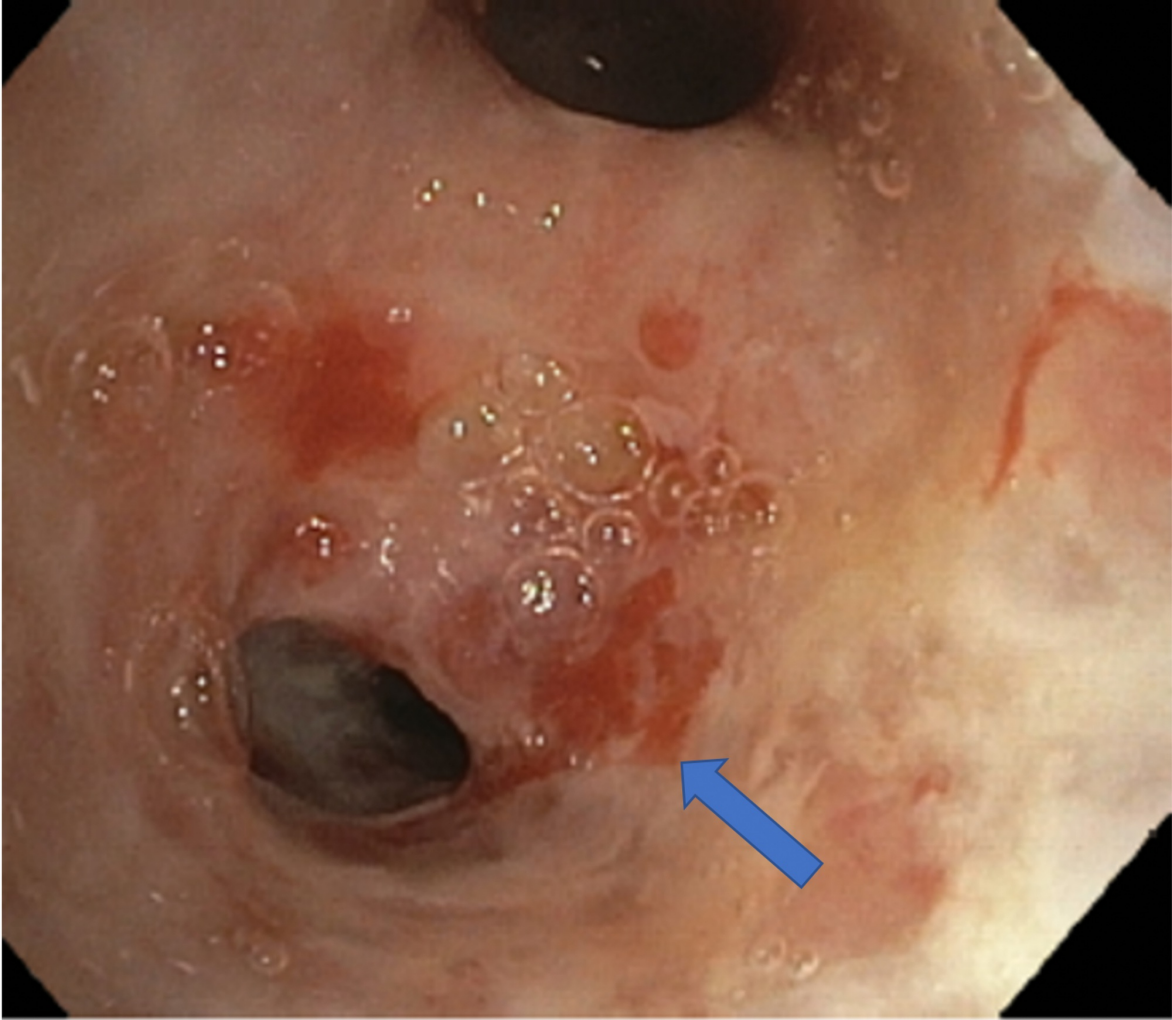
Bronchoscope imaging showing narrowing of the segmental bronchus and hyperemic lesions seen on the mucosa.

Subsequently, she underwent bronchoscopy as an out-patient to evaluate for suspected malignancy. Bronchoscopy revealed diffuse inflammation with edema of supraglottic mucosa (Figure 4), normal vocal cords, hyperemic tracheal mucosa, an ulcerative lesion on the first carina, and narrowing of multiple segmental bronchi secondary to inflamed edematous mucosa (Figures 5, 6).

QuantiFERON test was positive. Polymerase chain reaction (PCR) on bronchoscope sputum specimen confirmed Mycobacterium tuberculosis. Endobronchial biopsy from the left main bronchus also demonstrated reactive changes and necrotizing granulomatous inflammation. All of the sputum samples grew sensitive to Mycobacterial tuberculosis. She was promptly admitted to the hospital for the initiation of the four-drug regimen.

Upon presentation to the hospital, decreased breath sounds were appreciated bilaterally with prominent rhonchi. Initial blood tests including white blood cell count were unremarkable. Human immunodeficiency virus antibody-antigen testing was negative. Two induced sputum specimens were positive for acid-fast bacilli. She was promptly started on RIPE therapy (daily rifampin 600 mg, isoniazid 300 mg, pyrazinamide 1000 mg, ethambutol 800 mg, vitamin B6 50 mg) and was discharged home with home quarantine for the duration of her tuberculosis therapy after clearance with the Department of Health and Infection Control. Close contacts were preemptively treated.

## Discussion

This is the first case that is being reported of primary LTB with proximal spread to the tracheobronchial and pulmonary tuberculosis. LTB was one of the common forms of TB in the early 20th century, almost always occurred secondary to pulmonary tuberculosis. With the advent of antituberculous medications, the incidence of LTB had declined to 1-1.5% of reported tuberculosis [2, 3]. Laryngeal tuberculosis could be primary or secondary [4]. Primary LTB is solitary involvement of the larynx in the absence of pulmonary tuberculosis. Primary LTB is thought to begin with the direct inoculation of infectious Mycobacterium in the larynx. Primary LTB is reported in about 40% of LTB cases [5]. This is different from the development of secondary laryngeal TB that relies on either direct spread of bacilli from primary pulmonary TB or hematogenous seeding from an infectious nidus. The national tuberculosis surveillance system in the US collects data on the incidence and prevalence of TB. As per this large database, the most recent five years reported data is shown in Table 1.

**Table 1:** Number of cases of laryngeal tuberculosis (LTB) reported to National TB surveillance system.

Number of cases	2013	2014	2015	2016	2017
	4	4	3	2	0

Patients typically have non-specific presenting symptomatology and lacking apparent predisposing factors that delay diagnosis and treatment. Hoarseness and odynophagia are the most common presenting symptom, affecting 80-100% of patients [5-7]. Dysphagia, dyspnea, stridor, cough, and hemoptysis are other common complaints [8]. Middle-aged males are four times more likely to be affected than females [7]. A large number of modern cases do not have any clear identifiable risk factors such as immunodeficiency, malnutrition, or advanced age. There is also a historical shift favoring the involvement of the anterior larynx [3]. Vocal cords (50-70%) are the most affected site followed by the false cords (40-50%), epiglottis, subglottic region, and posterior commissure [9]. Biopsy of the primary growth itself is diagnostic and may show caseating granulomatous inflammation. Microbiological confirmation is generally low-yield.

Laryngoscopic and radiological findings of laryngeal TB resembles that of malignancy [6, 10, 11]. Direct visualization usually reveals hypertrophic, exophytic, and/or polypoid lesions. Physical findings vary but include laryngeal edema, hyperemia, nodularity, ulcerations, exophytic mass, and obliteration of anatomic landmarks [5, 12, 13]. In fact, there are growing concerns that radiographic similarities between TB and malignancy can delay their respective diagnoses [1]. CT and magnetic resonance imaging can better demonstrate the involvement of the surrounding structures and tissues of the larynx than does laryngoscopy [6]. Treatment is primarily with anti-tuberculous medications while surgery is reserved for cases of airway compromise. Laryngeal complications can occur. Thus, long-term follow-up is recommended.

Endobronchial tuberculosis (EBTB) is a tuberculous infection of the tracheobronchial tree supported by histological and microbiological evidence [14]. EBTB can affect any part of the bronchial tree. Five potential mechanisms of developing EBTB were suggested in the literature: (1) Direct extension from parenchymal focus, (2) spread through infected sputum, (3) hematogenous spread, (4) lymph node erosion into a bronchial tree, and (5) lymphatic spread [15]. It is interesting to note that in a recent study by Jung et al., more than 50% of patients with pulmonary tuberculosis are found to have EBTB [16]. Symptomatology varies widely depending on the site affected. Violent productive cough being the most common [17]. Bronchoscopy and computed tomography are the ideal modes of diagnosis. Sputum examination yields a variable diagnostic rate ranging from 16-53% [15, 18, 19]. Bronchoscopic appearance has been classified into seven subtypes: (1) nonspecific bronchitis, (2) hyperemic/edematous, (3) actively caseating, (4) granular, (5) ulcerative, (6) tumorous, and (7) fibrostenotic [20]. Stenosis is the most common complication despite adequate treatment. Treatment is similar to pulmonary tuberculosis.

In this case, while it is not clear if LTB or pulmonary tuberculosis is the primary inciting event leading to EBTB, we believe that our patient had primary LTB with direct spread to the tracheobronchial tree leading to EBTB further causing pulmonary parenchymal involvement. The CT imaging is consistent with primary pulmonary tuberculosis which does not explain her presenting complaints of dysphonia and hoarseness persistent for five months. If primary pulmonary tuberculosis was the inciting event, the patient’s symptomatology would have been different and we would be expecting tuberculoma formation with mediastinal lymphadenopathy in the five months of symptoms, which is not the case. The PET findings of the highest SUV in the larynx and the bronchoscopy findings support our hypothesis. This retrograde spread of LTB to EBTB and further to pulmonary tuberculosis has never been reported in the literature. LTB and EBTB are highly contagious and most infectious forms of tuberculosis [15].

## Conclusions

A high level of suspicion and early diagnosis can limit adverse complications and unnecessary exposure to healthcare workers. LTB should be suspected in patients presenting with hoarseness of voice, especially when associated with constitutional symptoms and necessary epidemiological measures taken to contain the spread.
